# Laryngeal Manifestations of Intractable Singultus

**DOI:** 10.7759/cureus.13730

**Published:** 2021-03-06

**Authors:** Jhon F Martinez Paredes, Chandler C Thompson, Amy L Rutt

**Affiliations:** 1 Otolaryngology - Head and Neck Surgery, Mayo Clinic, Jacksonville, USA

**Keywords:** intractable hiccups, singultus, vocal fold, granuloma, larynx

## Abstract

Hiccup is a common phenomenon experienced by almost everyone in life. Although the exact physiology of this phenomenon remains unknown, it is associated with multiple central and peripheral etiologic causes. Vocal fold granulomas are benign laryngeal lesions typically caused by iatrogenic trauma, voice misuse, or chronic irritation. We present, for the first time, an association between intractable hiccups and vocal fold granulomas with good response to acupuncture and voice therapy in a 62-year-old male patient. This is an important contribution to the literature as the first report describing the co-occurrence of these pathologies in the context of a patient with several treatment failures, including vagal nerve stimulator.

## Introduction

Hiccup is a common phenomenon experienced by almost everyone in life [1]. Nearly 4,000 hospitalizations a year in the United States can be attributed to this condition [2]. Singultus, derived from the Latin word “singult,” meaning “to catch one’s breath while sobbing,” is the medical term for hiccups [3]. It involves coordinated and involuntary contractions of the inspiratory muscles associated with a delayed and sudden closure of the glottis, which is responsible for the characteristic noise [1,4]. Some causes of hiccups related to the oropharynx and larynx are neck cysts, pharyngitis, laryngitis, esophagitis, esophageal lesions, and gastroesophageal reflux disease (GERD) [5]. Our case describes, for the first time, an association between intractable singultus and vocal fold granulomas in a male patient, suggesting an underlying pathophysiology that has yet to be elucidated.

## Case presentation

**Figure 1:**
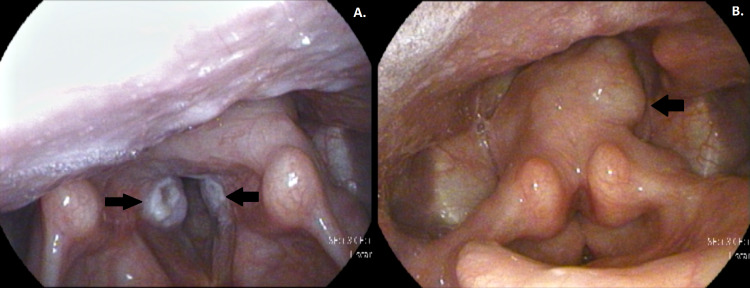
(A, B) Endoscopic view of bilateral vocal fold granulomas (see arrows) during flexible laryngoscopy.

A 62-year-old African-American man presented to the emergency department (ED) with inspiratory stridor, shortness of breath, and intractable hiccups. The patient is a retired veteran of the navy with a prior history of smoking and chronic obstructive pulmonary disease. He also had GERD and hiatal hernia, both surgically treated without relief of symptoms. Additionally, the patient was diagnosed with chronic persistent hiccups in 2017 and underwent vagal nerve stimulator (VNS) placement in 2018 without improving hiccups. The patient denied other comorbidities and reported that he was taking 20 mg omeprazole once daily. During his visit to the ED, physical examination and chest X-ray were negative for any pathology. Laboratory values were also unremarkable with a negative respiratory panel, including severe acute respiratory syndrome coronavirus 2. After the stridor and shortness of breath were resolved, the patient was evaluated at the laryngology outpatient clinic. He not only reported persistent hiccups but also trouble projecting his voice, singing, and swallowing according to the severity of hiccups and belching. During the flexible laryngoscopy and voice evaluation, paradoxical vocal fold movement (PVFM), muscle tension dysphonia, and bilateral posterior vocal fold granulomas were confirmed (Figure 1). The initial medical treatment included medical therapy for GERD, voice therapy sessions, and acupuncture.

Voice therapy strategies addressed PVFM, facilitating coordination in respiration and phonation as well as eliminating breath-holding tendency. The patient underwent 14 voice therapy/respiratory retraining sessions, with eight sessions occurring before restarting the VNS and six sessions occurring after. During the initial course of therapy, the incidence of hiccups decreased from 50 in 30 minutes to only five in 40 minutes, but this varied from week to week. By session eight, hiccups increased to 40 times in 40 minutes. The VNS was set at 0.50 ms without stridor, and voice therapy was resumed. During the subsequent six sessions of voice therapy, the incidence of hiccups occurred as follows: 15, three, 50, seven, five, and 30 times, with the patient and his wife noting “involuntary vocalizations” by the last session. The VNS was turned down to 0.25 ms, but the patient continued to have intractable hiccups. The patient noted improvement mainly with voice therapy and acupuncture. Voice therapy strategies included rescue breathing techniques, habitual tongue resting posture, postural adjustment, and strategies for jaw release. Additionally, other exercises involved semi-occluded vocal tract exercises, pitch glides, and singing to allow different coordination of respiration and phonation versus his habitual speaking mechanics. The patient was followed up for nine months by our laryngologist (A.L.R) and speech-language pathologist (C.C.T), with significant improvement of intractable hiccups. However, further speech therapy sessions were indicated to maintain control of symptoms.

## Discussion

An intractable singultus is characterized by hiccups that are present for 30 days or more [6]. If present, this condition can adversely impact mental and physical health and the patient’s quality of life [7]. Some consequences include sleep deprivation, mood disorders, aspiration and pneumonia, choking, malnutrition, impaired wound healing (in a postoperative context), or even death in an extreme condition [2]. Although the exact physiology of this phenomenon remains yet to be found, it is associated with multiple central and peripheral etiologic causes [1,5,8]. As mentioned before, some peripheral causes of singultus related to the oropharynx and larynx include cysts, tumors, or esophageal lesions; infectious scenarios involving the pharynx, larynx, and esophagus; and chronic conditions such as GERD [5]. Interestingly, no previous reports have described an association of intractable singultus with bilateral vocal fold granulomas.

Vocal fold granulomas are benign laryngeal lesions with a male predominance, typically caused by iatrogenic trauma, voice misuse, or chronic irritation [9]. Diagnosis of these lesions usually involves visualization of an ulcerated or nodular polypoid lesion in the vocal folds during flexible laryngoscopy [10]. It is well known that vocal fold granulomas can be secondary to continuous trauma or irritation [9]. Constant phonotrauma secondary to persistent coughing has been associated with the formation of these lesions in the posterior processes of the vocal folds [10,11].

Medical approaches for vocal fold granulomas include surgical and conservative therapy. However, several studies have shown that conservative therapy focused on eliminating vocal abuse and controlling the underlying cause of phonotrauma or irritation should be considered over surgery [12,13]. Suggested treatments for hiccups may vary according to the specific cause and include food restriction, physical maneuvers, behavioral therapy, pharmacological therapy, voice therapy, acupuncture, and interventional treatments as VNS and phrenic nerve blockade [5,6,14,15]. Noteworthy, a recent systematic review by Li et al. highlighted the role of acupuncture as a neuromodulation-like regulator among patients with chronic hiccups [15].

Our case report involves the co-occurrence of two different pathologies with underlying pathophysiology that has yet to be elucidated. Although GERD and voice overuse can be occasionally related to vocal fold granulomas, the location of the lesions in the posterior processes of the vocal folds leads us to consider a different feasible hypothesis based on the pathogenesis of vocal fold granulomas in the context of chronic cough [11]. We hypothesize that persistent phonotrauma secondary to the repetitive sudden closure of the glottis during hiccups episodes could lead to the formation of the granulomas found during flexible laryngoscopy. As mentioned above, intractable hiccups present in our patient impacted his quality of life, personal life, and mental health despite treatment with the VNS. This correlates with previous reports of intractable hiccups without response to VNS, requiring a multidisciplinary approach [16]. Therefore, other therapies, including acupuncture for neuromodulation, and voice therapy to facilitate coordination in respiration and phonation and eliminate breath-holding tendency have been indicated.

## Conclusions

An intractable singultus can adversely impact mental and physical health and the patient’s quality of life. We presented a case report of intractable singultus associated with bilateral vocal fold granulomas with good response to acupuncture and voice therapy. A proper and effective medical treatment of singultus should be the keystone in similar clinical scenarios. This is an important contribution to the literature as the first report describing the co-occurrence of these pathologies in the context of a patient with several treatment failures, including VNS.
